# Incidence and outcome of weaning from mechanical ventilation in medical wards at Thammasat University Hospital

**DOI:** 10.1371/journal.pone.0205106

**Published:** 2018-10-04

**Authors:** Narongkorn Saiphoklang, Jeerayuth Auttajaroon

**Affiliations:** Division of Pulmonary and Critical Care Medicine, Department of Medicine, Faculty of Medicine, Thammasat University, Pathumthani, Thailand; Azienda Ospedaliero Universitaria Careggi, ITALY

## Abstract

**Background:**

Weaning from mechanical ventilation is classified as simple, difficult, or prolonged according to weaning process. Theoretically, simple weaning group usually has better clinical outcomes than non-simple group; however, the results of previous studies were still inconsistent.

**Objectives:**

The purpose of the study was to determine the incidence, predictors, and outcomes of ventilator weaning and causes of weaning failure.

**Methods:**

A prospective observational study was performed between June and December 2013 in all patients (n = 164) who required mechanical ventilation with endotracheal intubation in medical wards at Thammasat University Hospital, Thailand. Duration of weaning, causes of weaning failure, extubation, reintubation, tracheostomy, number of ventilator-free days within 28 days, length of hospital stay, and hospital mortality were measured.

**Results:**

103 patients were eligible for final analysis. Mean ± SD age was 65.1±17.5 years and 55.3% were males. The incidences of simple, difficult and prolonged weaning were 46.6%, 36.9% and 16.5%, respectively. The mortality rates for simple, difficult, and prolonged weaning were 0%, 10.5% and 23.5% (p = 0.006), respectively. The 3 causes of weaning failure in non-simple weaning were bronchospasm, pneumonia, and malnutrition.

**Conclusions:**

Non-simple weaning increased mortality. Bronchospasm, pneumonia, and malnutrition were key risk factors for weaning failure. Strategies are needed to minimize their effects.

## Introduction

Critically ill medical patients often require mechanical ventilation to support their declining respiratory function. The most common clinical indications in the intensive care unit (ICU) setting are acute respiratory failure, coma, and neuromuscular disease [1]. The objectives of mechanical ventilation are to save the patient with acute severe hypoxemia or worsening respiratory acidosis, and can be used to relieve other respiratory symptoms [2]. Physicians alter ventilator settings appropriate to the patient’s clinical state and how it evolves. Weaning failure is an important issue in critically ill patients because it may result in increased morbidity (e.g. ventilator-associated pneumonia), hospital stay and mortality [3, 4].

In 2007, several European and American respiratory/intensive care societies held an international conference on weaning from mechanical ventilation and issued guidelines on weaning [4]. The SBT was identified as the major diagnostic test to determine whether patients could be successfully extubated. Weaning from mechanical ventilation was categorized into three groups: (1) simple weaning was defined as patients who were successfully weaned and extubated on the first attempt without difficulty, (2) difficult weaning was defined as patients who failed initial weaning and required up to three SBTs or needed up to 7 days from the first SBT to achieve successful weaning, and (3) prolonged weaning was defined as patients who failed at least three weaning attempts or require at least 7 days of weaning after the first SBT [4]. In most studies, weaning failure is defined as either the failure of SBT or the need for re-intubation within 48 hours following extubation [5, 6]. The causes of weaning failure are categorized by physiological system, i.e., respiratory, cardiovascular, neurological, neuropsychological, metabolic, nutritional causes, malnutrition, and anemia [4].

Data on weaning categories have been reported from medical and surgical ICUs in Austria. The rates of simple, difficult and prolong weaning were 59%, 26% and 14%, respectively. Hospital mortality increased in patients with prolonged (32%) but not difficult (9%) weaning in comparison with those with simple weaning (13%, p = 0.02) [7].

There is no weaning data from Thailand using the internationally recommended guidelines. In the study reported herein, we aimed to determine the incidence, predictors and outcomes of ventilator weaning, and the causes of weaning failure according to the new categories.

## Methods

### Study design

From June 2012 to December 2012, a prospective observational study was conducted in the medical unit at Thammasat University Hospital, a 540-bed tertiary care teaching hospital in the northern Bangkok conurbation, Thailand. The wards were medical ICU (8 beds), cardiac care unit (8 beds), stroke unit (6 beds), and 2 general medical wards (30 beds/ward). All patient care units were provided with the same standardized protocols of care for mechanically ventilated patients including secretion management, sedation protocol and ventilator-associated pneumonia prevention.

Ethics approval was obtained from the Ethics Committee of Faculty of Medicine, Thammasat University, Thailand (IRB No.MTU-EC-IM-0-083/56). Informed consent was obtained from all individual participants included in the study. The consent obtained from parents of the minors whose aged < 18 years.

### Study participants

Participants included patients who required mechanical ventilation and were over 15 years of age. Patients who died before or during weaning, who were transferred to another ward or another hospital, who had an unplanned extubation before or during weaning (e.g., accidental or self extubation), or who underwent a tracheostomy before or during weaning were excluded from the study.

### Baseline patient data

The collected data included sex, age, co-morbid diseases, illness severity measured by the Acute Physiology and Chronic Health Evaluation (APACHE) II score, admission ward, indication for intubation, weaning, causes of weaning failure, extubation, reintubation, tracheostomy, number of ventilator-free days within 28 days [defined as number of days alive without mechanical ventilation in the first 28 days or for subjects who die during mechanical ventilation, ventilator-free days were equal to 0], length of hospital stay, and hospital mortality.

The weaning process was under the supervision of the attending physicians. However, all members of the team who participated in the weaning process underwent pre-study training on weaning according to the consensus recommendations [4]. Briefly, physicians and nurses in charge (no licensed respiratory therapist in our country) were trained in two days with classes and workshops of mechanically ventilated patient care conducted by a pulmonary and critical care team that included five pulmonary and critical care physicians, two critical care nurses, and two medical equipment technicians. Weaning was conducted according to the standards of the European and American respiratory/intensive care societies [4] and considered as early as possible during the patients’ illness with a two-step approach in which readiness for weaning was assessed daily according to the criteria in the statement [4]. Patients who fulfilled these criteria underwent a SBT. The initial SBT last at least 30 minutes to 120 minutes and consisted of either breathing with a T-piece or a weaning trial undergoing 5–8 cmH_2_O pressure support with 5 cm H_2_O positive end-expiratory pressure. When patients successfully passed the SBT, the physician in charge, in collaboration with the attending medical staff initiated the weaning process. When a patient failed the initial SBT, mechanical ventilation was reinstituted and the physician reviewed the possible reversible causes of the weaning failure, including (1) respiratory factors e.g. bronchospasm, pulmonary edema, increased airway secretion, (2) cardiovascular factors e.g. congestive heart failure, myocardial infarction, (3) psychoneurologic factors e.g. sedative medication, delirium, and depression, (4) metabolic factors, e.g., electrolyte imbalances, dysglycemia, (5) nutritional factors, e.g., malnutrition and anemia. The SBT was repeated on the next day, if the patient was again ready to wean. A patient was rated as successfully weaned when he or she was extubated and breathing spontaneously without any invasive or noninvasive ventilatory support for ≥ 48 hours. Patients who had tracheostomies after simple weaning failure, were considered successfully weaned when they were breathing spontaneously, either through the tracheal cannula or directly through the tracheostomy, for 48 hours without any support. The total weaning duration was calculated as the days between the time when a patient first attempted initial SBT and the time when a patient was successfully weaned from mechanical ventilation for the last time.

### Definitions

Weaning failure was defined as either the failure of SBT or the need for reintubation within 48 hours following extubation [4]. Participants were classified into 3 groups: (1) simple weaning: successful weaning and extubation on the first attempt without difficulty, (2) difficult weaning: failure of initial weaning and the need for up to three SBTs for as many as 7 days from the first SBT to achieve successful weaning; and (3) prolonged weaning: failure of at least three weaning attempts or the need for at least 7 days of weaning after the first SBT [4].

### Outcome measures

The primary outcome measure was the number of patients in each category. The secondary outcomes were hospital mortality, rate of self-extubation and unplanned extubation, re-intubation rate, tracheostomy rate, number of ventilator-free days within 28 days and length of hospital stay. The possible factors that contributed to weaning failure were broadly categorized by physiological system [4] and included: (1) respiratory: bronchospasm, pneumonia, pulmonary edema, increased airway secretions; (2) cardiovascular: acute myocardial infarction and congestive heart failure; (3) neurological: acute stroke, muscle weakness, and sedative or hypnotic medications; (4) neuropsychological: delirium, anxiety and depression; (5) metabolic: metabolic alkalosis, hypoglycemia or hyperglycemia, hypokalemia (serum potassium level < 3.5 mg/dL), hypomagnesemia (serum magnesium level < 1.8 mg/dL), hypophosphasemia (serum phosphate level < 2.5 mg/dL), and steroid use; (6) nutritional: malnutrition [body mass index (BMI) < 20 kg/m^2^, or serum albumin level < 3.5 g/dL], overweight (BMI > 25 kg/m^2^), and (7) anemia defined as hemoglobin concentration < 8 g/dL. Factors causing weaning failure were diagnosed by physicians in charge at the time of the first SBT failure.

### Statistical analysis

Based on a previous study [7], we calculated that a sample size of 185 patients would provide a power of 80% at a significance level of 0.05 to determine a significant between-group difference of at least 20% in the primary outcome. Chi-squared test was used to compare categorical variables between the three weaning categories or two groups. Kruskal-Wallis or ANOVA tests were used for the comparison of continuous variables. Multivariable logistic regression analysis was used to explore the risk factors for weaning failure (i.e. difficult and prolonged weaning vs. simple weaning) using a backward-stepwise selection. Data are presented as means ± SD, medians (interquartile ranges), or proportions, as appropriate. A two-sided p-value < 0.05 was considered statistically significant. Statistical analyses were performed using SPSS version 16.0 software (SPSS Inc., Chicago, IL, USA).

## Results

**Fig 1:**
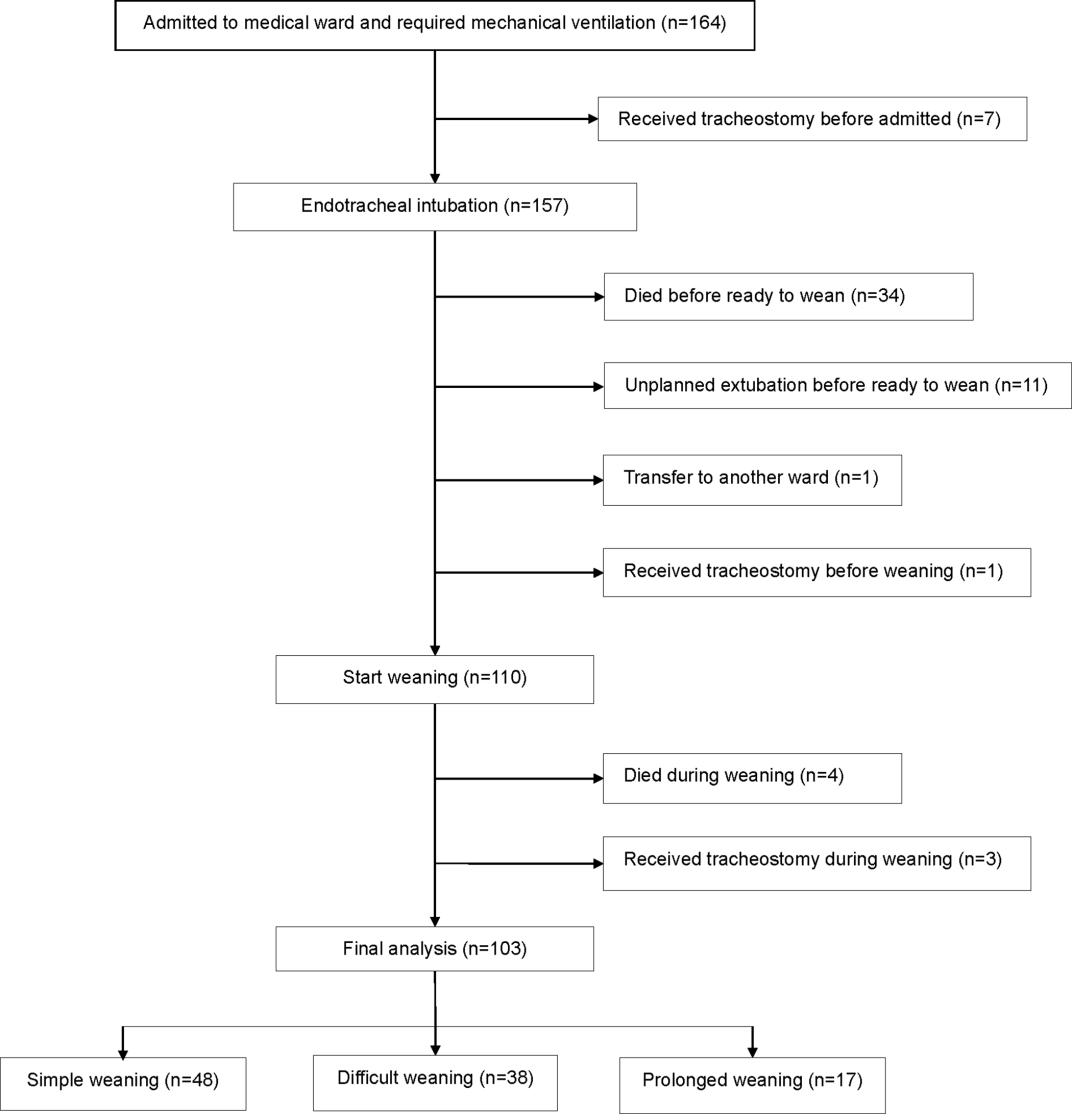
Study flowchart indicates inclusion and exclusion population.

One hundred sixty-four mechanically ventilated patients were screened and 103 of these were included in the final analysis (Fig 1). Mean ± SD age was 65.1±17.5 years and 55.3% were males.

The baseline characteristics of the sample are shown (Table 1) and did not differ between the groups. The rates of simple, difficult and prolong weaning were 48 (46.6%), 38 (36.9%) and 17 (16.5%), respectively. The prolonged weaning group had the worst morbidity and mortality outcomes and certain outcomes increased significantly from simple to difficult to prolonged weaning (Table 2). Bivariate analyses identified a number of significant factors (mostly respiratory and cardiovascular) for weaning failure (Table 3).

**Table 1:** Baseline characteristics of the 103 patients who started weaning.

Data	Simple weaning n = 48	Difficult weaning n = 38	Prolonged weaning n = 17	p-value
**Age**, years	70.5 (52.0–74.8)	70.5 (61–77.5)	70.0 (57.5–77.5)	0.989
**Males**	31 (64.6)	18 (47.4)	8 (47.1)	0.212
**Types of comorbidity**				
Malignancy	0 (0)	3 (7.9)	1 (5.9)	0.156
Chronic kidney disease	11 (22.9)	9 (23.7)	2 (11.8)	0.576
Chronic respiratory failure	6 (12.5)	7 (18.4)	5 (29.4)	0.289
Chronic heart failure	7 (14.6)	5 (13.2)	2 (11.8)	0.955
Diabetes mellitus	19 (39.6)	10 (26.3)	8 (47.1)	0.255
HIV infection	1 (2.1)	1 (2.6)	0 (0)	0.805
**APACHE II score**, points	12.5 ± 5.5	13.5 ± 4.9	16.3 ± 7.0	0.295
**Predicted mortality assessed by APACHE II**, %	16.5 (9.9–23.5)	14.9 (11.8–26.9)	21.0 (13.4–42.4)	0.155
**Indications for MV**				0.758
Respiratory disease	28 (58.3)	22 (57.9)	13 (76.5)	
Cardiovascular disease	6 (12.5)	5 (13.2)	2 (11.8)	
Neurological disease	5 (10.4)	6 (15.8)	1 (5.9)	
Airway protection	9 (18.8)	5 (13.2)	1 (5.9)	
**Admission wards**				0.205
General wards	36 (75.0)	25 (65.8)	16 (94.1)	
Cardiac care unit	5 (10.4)	2 (5.3)	0 (0)	
Medical intensive care unit	5 (10.4)	5 (13.2)	1 (5.9)	
Stroke unit	2 (4.2)	6 (15.8)	0 (0)	
**Types of MV**				0.054
Assist-control respirator	21 (43.8)	26 (68.4)	11 (64.7)	
Bird respirator	27 (56.2)	12 (31.6)	6 (35.2)	
**Modes of SBT for weaning**				0.543
PS with PEEP	5 (10.4)	7 (18.4)	2 (11.8)	
T-piece	43 (89.6)	31 (81.6)	15 (88.2)	
**Duration of SBT**, minutes	120 (120–120)	120 (120–150)	135 (120–150)	0.063

Data are presented as n (%), mean±SD and median (interquartile range), unless otherwise stated. HIV = human immunodefiency virus, APACHE II = Acute Physiology and Chronic Health Evaluation II, MV = mechanical ventilation, SBT = spontaneous breathing trial, PS = pressure support, PEEP = positive end-expiratory pressure

**Table 2:** Incidence and outcome for the 103 patients who started weaning.

Data	Simple weaning n = 48	Difficult weaning n = 38	Prolonged weaning n = 17	p-value
Duration of intubation, days	2.0 (1.0–4.0)^a^	6.0 (3.8–9.0)^b^	13.0 (7.0–27.0)^c^	<0.001
Weaning duration, days	0 (0–0) ^a^	1.0 (1.0–2.0)^b^	4.0 (3.0–7.5)^c^	<0.001
Duration from first intubation to tracheostomy, days	0	24.0 ± 6.2	29.5 ± 7.8	0.375
Duration from tracheostomy to discharge, days	0	58.3 ± 74.4	34.5 ± 14.7	0.555
Re-intubation rate	0 (0)^a^	7 (18.4)^b^	4 (23.5)^c^	0.004
Tracheostomy rate	0 (0)^a^	3 (7.9)^b^	4 (23.5)^c^	0.004
Number of ventilator-free days within 28 days	25.0 (23.5–26.0)^a^	20.0 (13.5–22.0)	13.0 (0–20.0)^c^	<0.001
Hospital length of stay, days	12.0 (5.5–20.5)	17.0 (14.0–25.0)^b^	26.0 (20.0–42.0^c^	0.001
Hospital mortality rate	0 (0) ^a^	4 (10.5)^b^	4 (23.5)^c^	0.006

Data are presented as n (%), mean±SD and median (interquartile range), unless otherwise stated.

a : Significant difference for simple vs. difficult weaning (p<0.05)

b : Significant difference for difficult vs. prolonged weaning (p<0.05)

c : Significant difference for prolonged vs. simple weaning (p<0.05)

**Table 3:** Causes of weaning failure*.

Factors	Simple weaning n = 48	Difficult and prolonged weaning n = 55	p-value
**Respiratory problem**			
Bronchospasm	2 (4.2)	26 (47.3)	<0.001
Pneumonia	1 (2.1)	23 (41.8)	<0.001
Pulmonary edema	0 (0)	10 (18.2)	0.002
Increased airway secretion	1 (2.1)	21 (38.2)	<0.001
**Cardiovascular problem**			
Increased oxygen demand from sepsis	0 (0)	7 (12.7)	0.014
Congestive heart failure	0 (0)	9 (16.4)	0.003
Acute myocardial infarction	0 (0)	2 (3.6)	0.503
**Neurological problem**			
Stroke	0 (0)	4 (7.3)	0.122
Muscle weakness	0 (0)	0 (0)	1
Sedative or hypnotic medications	0 (0)	0 (0)	1
**Psychological problem**			
Delirium	0 (0)	0 (0)	1
Depression	0 (0)	2 (3.6)	0.502
**Metabolic problem**			
Metabolic alkalosis	0 (0)	3 (5.5)	0.256
Hyperglycemia	1 (2.1)	7 (12.7)	0.065
Hypokalemia	1 (2.1)	6 (10.9)	0.122
Hypomagnesemia	1 (2.1)	4 (7.3)	0.373
Hypophosphatemia	0 (0)	2 (3.6)	0.503
Steroid use	0 (0)	5 (9.1)	0.059
**Nutritional problem**			
Malnutrition	1 (2.1)	26 (47.3)	<0.001
Overweight	0 (0)	1 (1.8)	1
Anemia	2 (4.2)	19 (34.5)	<0.001

Data are presented as n (%).

*Factors causing weaning failure were diagnosed by physicians in charge at the time of the first SBT failure.

Bronchospasm, pneumonia, and malnutrition were identified as independent explanatory variables for weaning failure when tested in the multivariate model (Table 4).

**Table 4:** Multivariate logistic regression analysis for causes of weaning failure in difficult and prolonged weaning compared with simple weaning.

Factors	Odds ratio	95%CI	P-value
Bronchospasm	9.1	1.6–50.0	0.012
Pneumonia	14.8	1.6–138.5	0.018
Malnutrition	29.5	3.4–254.7	0.002

There were no significant differences in clinical outcomes between patient admitted in intensive units (ICU/CCU/stroke unit) or general wards although some baseline characteristics were significantly different (S1 and S2 Tables).

## Discussion

This prospective observational study is the first study from Thailand on ventilator weaning and has included patients admitted to both general medical wards and ICU. The health care system in our country, especially in our hospital, differs from many developed countries because of various limitations of healthcare resources and well-trained personnel. Healthcare costs are covered by the Ministry of Public Health in Thailand for any adults (i.e., aged over 15 years) and any children admitted to government hospitals. However, insufficient ICU availability and limited government spending has led to many critically ill patients being admitted to general wards rather than intensive care units, leading to inadequate patient treatment and monitoring. These reasons may have influenced the study outcomes.

Although the 2007 weaning classification is widely acceptable but this category was discussed in light of the weaknesses highlighted by the WIND study [8] and of MacIntyre editorial [9]. The 2007 weaning definition could not classify almost half of invasively ventilated patients from this mixed medical and surgical ICU population [4]. Fortunately, the WIND study proposed the simple definition based on the concept of separation attempts and mainly on the duration of the weaning process after a first attempt. There were 4 groups including group no weaning process, group 1 (short weaning), group 2 (difficult weaning), and group 3 (prolonged weaning). Those showed mortality rate in 86%, 5.8%, 16.5% and 29.8%, respectively. Median duration from the first separation attempt to success in group 1, 2 and 3 were 0, 3 and 11 days, respectively.

We identified the incidence and outcome of weaning, and the causes of weaning failure according to the 2007 designated categories [4]. The main finding was that the incidence of simple weaning was the most common weaning outcome, being three times and 10% more common than prolonged and difficult weaning, respectively. Similar rates for these weaning groups have been reported in 257 patients in Austria by Funk et al [7], namely, 59%, 26% and 14%, respectively. However, in France, Tonnelier et al [10] reported different weaning classification rates of 29.5%, 41% and 29.5%, respectively, in 115 patients. The lower incidence rate of simple weaning reported in the latter study may be due to the subject population, as only patients who required mechanical ventilation for more than 48 hours were enrolled. The difference in outcomes reported may also be because post-operative surgical patients are usually weaned more easily than medical patients. Both studies [7, 10] recruited medical and surgical patients, differing from our study enrolling only medical patients.

We identified bronchospasm, pneumonia, and malnutrition as significant risk factors for failed weaning. These factors were considered as important key factors of weaning failure in weaning protocols [11]. Other studies have found similar results, as well as other factors not identified in our study. Huang et al [12] and Beuret et al [13] found ineffective cough was the best predictor of extubation failure. Another study by Dalar et al [14] showed tracheal stenosis led to extubation failure and prolonged mechanical ventilation. Thille et al [15] found underlying chronic cardiac or respiratory comorbidities as important risk factors for extubation failure in patients aged > 65 years; these comorbidities were also identified by Funk et al [7]. By contrast, our study did not identify any particular comorbidity.

The 2007 weaning classification did not correlate with the illness severity [defined by the simplified acute physiology score (SAPS) II score] in Funk’s study [7] and Tonnelier’s study [10]. Nor did the SAPS II score predict mortality. Similar to the results found in our study, the APACHE II score was not able to predict weaning failure.

Our hospital mortality rate significantly increased with increasing severity of weaning category (0% to 10.5% to 23.5%, respectively). Funk’s study [7] reported a similar trend: 13%, 9%, and 32% (p = 0.02), respectively, but only prolonged weaning was independently associated with an increased risk of death.

We also experienced increasing reintubation rates with increasing severity of weaning classification. Funk found a reverse trend but this was not significantly different [13%, 7% and 5% (p = 0.18)], and Tonnelier had a similar trend to our study but again this was not statistically significant: 0%, 6% and 15%, respectively, (p = 0.24). Similarly, our tracheostomy rates increased in parallel to weaning severity, consistent with Funk’s data. However, he recorded rates twice as high (68%) in his prolonged weaning patients, which may have been due a greater proportion of patients with chronic respiratory failure. Prolonged weaning was associated with an overall increase in hospital stay in our study and those of Funk and Tonnelier.

There are some limitations of the study. Firstly, the sample size was small and this limited our statistical power to detect differences in some parameters and leading to the wide confidence intervals. Secondly, no data was collected on ventilator parameters. Finally, we cannot exclude the possibility that the variation in physician decisions may have biased our results, though we attempted to minimize this bias by providing pre-study training on the weaning recommendations.

## Conclusions

Non-simple weaning, especially prolonged weaning was associated with increased mortality, re-intubation and tracheostomy rates, length of hospital stay, and decreased ventilator-free days. Key risk factors for weaning failure were bronchospasm, pneumonia, and malnutrition. Strategies are needed to counter the deleterious effects of these risk factors.

## Supporting information

S1 Table Baseline characteristics of the 103 patients comparing between general wards and intensive units. (DOCX)

S2 Table Incidence and outcome for the 103 patients comparing between general wards and intensive units. (DOCX)
