# Supplementary material for: Incidence and outcome of weaning from mechanical ventilation in medical wards at Thammasat University Hospital
Source: PLoS One. 2018 Oct 4;13(10):e0205106. doi: 10.1371/journal.pone.0205106 (PMC6171918; doi:10.1371/journal.pone.0205106)
Supplement: S1 Table — (DOCX) [file pone.0205106.s001.docx]

**S1 Table.** Baseline characteristics of the 103 patients comparing between general wards and intensive units^a^

| **Data** | **General wards**  **n=77** | **Intensive units^a^**  **n=26** | **p-value** |
| --- | --- | --- | --- |
| **Age**, years | 66.2 ± 17.4 | 62.0 ± 17.7 | 0.302 |
| **Males** | 37 (48.1) | 20 (76.9) | 0.010 |
| **Types of comorbidity** |  |  |  |
| Malignancy | 2 (2.6) | 2 (7.7) | 0.264 |
| Chronic kidney disease | 14 (18.2) | 8 (30.8) | 0.176 |
| Chronic respiratory failure | 17 (22.1) | 1 (3.8) | 0.026 |
| Chronic heart failure | 11 (14.3) | 3 (11.5) | 0.508 |
| Diabetes mellitus | 31 (40.3) | 6 (23.1) | 0.114 |
| HIV infection | 2 (2.6) | 0 (0) | 0.557 |
| **APACHE II score**, points | 13.2 ± 5.5 | 14.4 ± 6.3 | 0.393 |
| **Predicted mortality assessed by APACHE II**, % | 19.8 ± 11.4 | 22.5 ± 16.8 | 0.452 |
| **Indications for MV** |  |  | 0.059 |
| Respiratory disease | 66 (85.7) | 15 (57.7) |  |
| Cardiovascular disease | 7 (9.1) | 7 (26.9) |  |
| Neurological disease | 5 (6.5) | 7 (26.9) |  |
| Airway protection | 13 (16.9) | 3 (11.5) |  |
| **Types of MV** |  |  | <0.001 |
| Assist-control respirator | 33 (42.9) | 25 (96.2) |  |
| Bird respirator | 44 (57.1) | 1 (3.8) |  |
| **Modes of SBT for weaning** |  |  | 0.254 |
| PS with PEEP | 9 (11.7) | 5 (19.2) |  |
| T-piece | 68 (88.3) | 21 (80.8) |  |
| **Duration of SBT**, minutes | 131.2 ± 52.8 | 133.4 ± 41.8 | 0.822 |

Data are presented as n (%) and mean ± SD, unless otherwise stated. HIV=human immunodefiency virus, APACHE II=Acute Physiology and Chronic Health Evaluation II, MV= mechanical ventilation, SBT=spontaneous breathing trial, PS=pressure support, PEEP=positive end-expiratory pressure

^a^ include medical intensive care unit, cardiac care unit and stroke unit
