# Supplementary material for: Incidence and outcome of weaning from mechanical ventilation in medical wards at Thammasat University Hospital
Source: PLoS One. 2018 Oct 4;13(10):e0205106. doi: 10.1371/journal.pone.0205106 (PMC6171918; doi:10.1371/journal.pone.0205106)
Supplement: S2 Table — (DOCX) [file pone.0205106.s002.docx]

**S2 Table**. Incidence and outcome for the 103 patients comparing between general wards and intensive units^a^

| **Data** | **General wards**  **n=77** | **Intensive units^a^**  **n=26** | **p-value** |
| --- | --- | --- | --- |
| Duration of intubation, days | 6.9 ± 7.5 | 15.6 ± 36.7 | 0.241 |
| Weaning duration, days | 1.7 ± 3.3 | 0.7 ± 0.9 | 0.021 |
| Duration from first intubation to tracheostomy, days | 25.0 ± 4.9 | 6.0 ± 0 | 0.057 |
| Duration from tracheostomy to discharge, days | 43.2 ± 50.2 | 54.0 ± 0 | 0.850 |
| Re-intubation rate | 10 (13.0) | 1 (3.8) | 0.176 |
| Tracheostomy rate | 6 (7.8) | 1 (3.8) | 0.432 |
| Number of ventilator-free days within 28 days | 18.6 ± 8.7 | 19.7 ± 7.2 | 0.543 |
| Hospital length of stay, days | 23.2 ± 26.2 | 23.1 ± 23.1 | 0.118 |
| Hospital mortality rate | 8 (10.4) | 0 (0) | 0.089 |

Data are presented as n (%) and mean ± SD.

^a^ include medical intensive care unit, cardiac care unit and stroke unit
